# Functional and Structural Correlates of Memory in Patients with Mesial Temporal Lobe Epilepsy

**DOI:** 10.3389/fneur.2015.00103

**Published:** 2015-05-13

**Authors:** Alexander J. Barnett, Min Tae M. Park, Jon Pipitone, M. Mallar Chakravarty, Mary Pat McAndrews

**Affiliations:** ^1^Department of Psychology, University of Toronto, Toronto, ON, Canada; ^2^Schulich School of Medicine and Dentistry, Western University, London, ON, Canada; ^3^Douglas Mental Health University Institute, Montreal, QC, Canada; ^4^Research Imaging Centre, Centre for Addiction and Mental Health, Toronto, ON, Canada; ^5^Department of Psychiatry and Biomedical Engineering, McGill University, Montreal, QC, Canada

**Keywords:** temporal lobe epilepsy, hippocampus, memory, fMRI, structural MRI

## Abstract

Individuals with medial temporal lobe epilepsy (mTLE) often show material-specific memory impairment (verbal for left, visuospatial for right hemisphere), which can be exacerbated following surgery aimed at the epileptogenic regions of medial and anterolateral temporal cortex. There is a growing body of evidence suggesting that characterization of structural and functional integrity of these regions using MRI can aid in prediction of post-surgical risk of further memory decline. We investigated the nature of the relationship between structural and functional indices of hippocampal integrity with pre-operative memory performance in a group of 26 patients with unilateral mTLE. Structural integrity was assessed using hippocampal volumes, while functional integrity was assessed using hippocampal activation during the encoding of novel scenes. We quantified structural and functional integrity in terms of asymmetry, calculated as (L − R)/(L + R). Factor scores for verbal and visual memory were calculated from a clinical database and an asymmetry score (verbal − visual) was used to characterize memory performance. We found, as expected, a significant difference between left and right mTLE (RTLE) groups for hippocampal volume asymmetry, with each group showing an asymmetry favoring the unaffected temporal lobe. Encoding activation asymmetry showed a similar pattern, with left mTLE patients showing activation preferential to the right hemisphere and RTLE patients showing the reverse. Finally, we demonstrated that functional integrity mediated the relationship between structural integrity and memory performance for memory asymmetry, suggesting that even if structural changes are evident, ultimately it is the functional integrity of the tissue that most closely explains behavioral performance.

## Introduction

Medial temporal lobe epilepsy (mTLE) is characterized by recurrent seizures generated in temporal lobe structures, specifically the hippocampus. The MTLs are known to be highly involved in episodic memory (1, 2) and, the disruption of neural circuitry seen in mTLE is accompanied by material-specific memory deficits (3). Individuals with left mTLE (LTLE) often present with verbal memory deficits, while those with right mTLE (RTLE) are more likely to show memory deficits for visuospatial material that is difficult to verbalize (4–6). Surgical intervention for mTLE involves resection of the anterior hippocampus, amygdala, and anterior temporal neocortex in the affected hemisphere to relieve seizures. This resection can result in a *de novo* material-specific memory decline or an exacerbation of pre-surgical weaknesses (7–9).

Standard clinical investigations aim to assess the integrity of MTL tissue before resection to provide an estimate for predicted change. Several key predictors have emerged as being important for understanding this change. First, pre-surgical memory performance has been shown to significantly predict post-operative change in that higher initial performance is associated with greater decline (7, 10–12). Second, individuals who have mesial temporal sclerosis (MTS) tend to show smaller declines in memory postoperatively compared to those without MTS. These individuals also tend to have worse memory presurgically, which may be attributed to their lesioned hippocampus and the atrophy in the surrounding tissue (4, 13, 14). A number of studies have recently applied task-related activation in fMRI to assess the integrity of medial temporal memory areas. Greater activation of the to-be-resected hippocampus has been correlated with a larger magnitude of post-operative memory decline (15–19); both magnitude and spatial extent have been used to quantify activation. Few studies have looked at all three of these predictors concurrently and, among those that have, there is disagreement regarding which predictor performs most effectively (15, 16).

As of yet, no study has investigated the relationship among these three factors and how they influence each other, which may shed light on inconsistency in the literature. One recent study by Bigras et al. (20) investigated the lateralization of MTL activity during a scene encoding task. They found that patients with LTLE who showed greater right than left MTL activation during scene encoding performed worse on concurrent neuropsychological tests of verbal memory. In patients with RTLE, those that showed greater left MTL activation during scene encoding performed worse on neuropsychological measures of visual memory. They also found that larger hippocampal volume was associated with greater peak activation in the affected hemisphere for both TLE groups, suggesting that structural integrity may constrain functional measures (20). What remains unclear is how the structural integrity (volume) and functional integrity (activation) are jointly contributing to behavioral performance. In a recent examination of the default mode network in epilepsy, we found that functional connectivity of the network was a critical mediator of the relationship between memory and brain structural integrity (21).

The aim of the current study was to extend our examination of the joint influences of structural and functional integrity by focusing on the MTL and pre-surgical measures of memory. In accordance with previous literature, we examined these neuroimaging measures with asymmetry ratios (ARs), which quantify the asymmetric contribution to a measure between the two hemispheres. As it has been shown in several other studies using the scene encoding paradigm, we predicted that structural and functional hippocampal ARs individually would be related to pre-surgical memory performance and also anticipated the two ARs would be correlated. Our novel prediction was that we would find a mediating relationship when these were jointly used to explain variance in memory performance, such that the influence of structure would be reduced when the contribution of functional activation is considered.

## Materials and Methods

### Participants

Twenty-six patients with pharmacologically intractable unilateral TLE were recruited from the epilepsy surgery program at Toronto Western Hospital. Fourteen presented with RTLE (7 men, 7 women; mean age = 36.2 years, range = 19–58 years) and 12 presented with LTLE (4 men, 8 women; mean age = 35.9 years, range = 19–53 years). Seizure focus was determined using scalp EEG, and (if necessary) intracranial EEG. Patients were classified as having mTLE based on a constellation of signs and symptoms including ictal semiology, EEG findings from inpatient monitoring, imaging, and neuropsychological profile, in accord with ILAE guidelines (21). A group of 12 healthy controls (8 men, 4 women; mean age = 29.4, range = 23–38) with no history of neurological disorders were recruited to provide an estimate of normal asymmetry for comparison. A summary of demographics can be found in Table 1. Informed consent was obtained from all participants in this study in accordance with the UHN Research Ethics Board.

**Table 1:** **Patient demographic data**.

	Controls	RTLE	LTLE
*N*	12	14	12
Age, y (SD)	29.4 (5.0)	36.2 (11.6)	35.9 (10.9)
Education, y (SD)	17.9 (4.3)	13.9 (3.8)	14.8 (2.8)
Sex, M/F	8/4	7/7	4/8
Handedness, R/L	11/1	12/2	11/1
Language dominance, R/L/BI	0/12/0	1/12/1	0/11/1
Disease duration, y (SD)	–	16.2 (14.2)	17.0 (11.8)
Onset of seizures, y (SD)	–	20.9 (13.5)	19.0 (16.0)
Presence of MTS, Yes/No	–	8/6	8/4
Other lesions	–	3	2
Verbal memory factor	–	0.11 (0.99)	−0.20 (1.3)
Visual memory factor	–	−0.24 (1.1)	0.59 (0.93)
IQ factor	–	0.11 (1.0)	0.38 (1.0)

*RTLE, right temporal lobe epilepsy; LTLE, left temporal lobe epilepsy; y, years; SD, standard deviation; M, male; F, female; R, right; L, left; BI, bilateral; IQ, intelligence quotient*.

*Characterization of MTS and other lesions was based on radiology (3 T MRI protocol). Three patients with RTLE showed right MTL cortical dysplasia, one with dual pathology of MTS. With respect to extra-MTL lesions, MRI demonstrated cortical dysplasia in the left posterior insula surrounding Heschl’s gyrus in one LTLE patient (with concomitant MTS) and a heterotopion in the left occipital horn in another LTLE patient. All patients were subsequently referred for standard anterior temporal lobe resections*.

### Neuropsychological testing

Patients were administered a comprehensive neuropsychological battery as part of a clinical pre-operative evaluation, which included several tests assessing learning/memory, language, and intelligent quotient (IQ). Verbal memory, visuospatial memory, and IQ scores were calculated based on a previously reported principal component analysis (PCA) performed from our clinical database (22). This analysis used PCA to reduce and summarize neuropsychological measures for a group of 56 pre-surgical patients with mTLE (28 RLTE, 28 LTLE). The three test scores for the verbal component included total score on the Warrington recognition memory test for words (23) and the total learning score and percent retention score on the Rey auditory verbal learning test (24). The component for visual memory was based on the total score for Warrington recognition memory test for faces (23), the total number of designs reproduced over five learning trials from the Rey visual design spatial conditional associative learning test (25), and the total number of trials to reach criterion for the spatial conditional associative learning task (26). The IQ component was based on verbal and performance IQ index scores from the Wechsler abbreviated scale of intelligence (27). In the current study, we *z*-transformed the patient cohorts’ test scores based on the distribution of scores from St-Laurent et al. (22) and, using the factor loading scores, computed three PCA scores for each participant reflecting verbal memory, visual memory, and IQ factors. For the purposes of the mediation analysis, we produced a memory asymmetry scores based on the formula (Verbal memory factor − Visual memory factor).

### fMRI data acquisition

Data were collected on a 3-T Signa MR system (GE Medical Systems, Milwaukee, WI, USA). A high-resolution 3D anatomic scan was collected first for visualization, normalization of fMRI data to a common anatomic template, and volumetric analysis (T1-weighted sequence, FOV 220 mm, 146 slices, flip angle = 12°, TE = 3 ms, TR = 8 ms, 256 × 256 matrix, resulting in voxel size of 0.85939 × 0.85939 × 1.0). Echo-Planar Imaging sequences (TE = 30 ms, TR = 2000 ms, voxel size 3.75 mm × 3.75 mm × 5mm, 32.5 mm oblique slices angled to be orthogonal to the long axis of the hippocampus to minimize partial volume effects in the MTL) were run during the 6½ min functional scan.

### fMRI task

Participants performed a commonly used scene encoding task during an fMRI scan. While in the scanner they viewed a series of 60 complex visual scenes for 3500 ms each. Two of these visual scenes were exposed repeatedly immediately prior to the fMRI run and were presented 15 times each during the critical run to serve as “baseline” trials while the other 30 images were novel. Participants were instructed to study the novel scenes for a later memory test, which was, in fact, not administered. Presentation order of scenes was randomized for each subject. Previous literature has demonstrated reliable hippocampal activation for a contrast of novel images > repeated images (28).

### Functional data preprocessing

Preprocessing was done using Statistical Parameter Mapping 8 (SPM8; Wellcome Department of Imaging, London) run through Matlab 7.9 (Mathworks, Inc.). The first three images of the functional scan were dropped to remove acquisition artifact. Preprocessing included realignment of anatomical and functional scans to the anterior commissure followed by co-registration of the functional scans to the anatomical scan. The functional scans were then realigned and unwarped to correct for scanner motion artifact and subject movement. The anatomical scan was segmented into gray matter, white matter, and cerebral spinal fluid and was used to calculate normalization parameters to MNI space. The resulting parameter files of this segmentation were then used to normalize functional and anatomical scans into MNI space using affine registration. Finally, the functional images were smoothed using a full-width at half maximum Gaussian smoothing kernel of 8 mm × 8 mm × 8 mm. Data went through a high-pass filter to account for low-frequency drift. Each stimulus event was then modeled by SPM8’s canonical hemodynamic response function.

### fMRI data analysis

**Figure 1:**
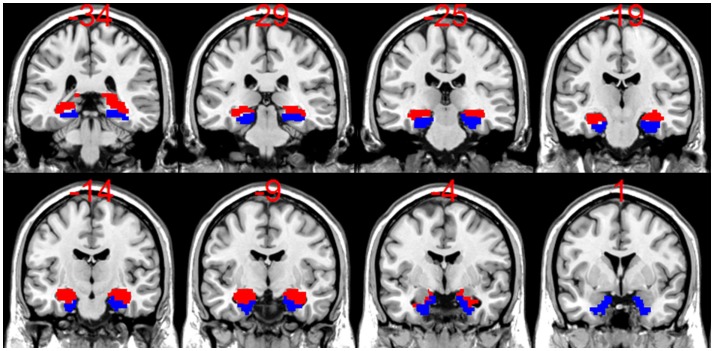
**A display of coronal slices from a template T1 anatomical scan from SPM8 overlaid with bilateral hippocampal (HC) mask (in red) and bilateral parahippocampal (PHC) masks (in blue), which were used to calculate the activation asymmetry ratios (ARs)**. Note that for the HC + PHC activation ARs, the bilateral HC mask and bilateral PHC mask were added together.

Novel encoding scenes were contrasted against repeated events using an event-related, fixed-effects, general linear model (GLM) in SPM8 for each subject. The resulting contrast images were used for group level, random-effects analyses to visualize activation for LTLE, RTLE, and healthy control groups. Hippocampal and parahippocampal masks made in MARINA (29)^1^ from the AAL atlas by Tzourio-Mazoyer et al. (30) were applied to each patient’s contrast image to find voxel counts and *t*-values at a threshold of *t* > 1. This liberal threshold was chosen to allow for an inclusive representation of activated voxels, while excluding those that are very likely to be due to chance. The application of low thresholding for this purpose has been applied in the past (16, 31) when both magnitude and spatial extent of activation are considered important. Individual masks were made for left and right hippocampus and parahippocampus as shown in Figure 1. We used a bilateral hippocampal mask and a bilateral hippocampal/parahippocampal mask to assess asymmetry. Using these masks, we extracted the voxel counts weighted by their *t*-value for positively activated voxels with the laterality index toolbox (32). These weighted voxel counts (WVC) were used to create activation ARs using the formula [(Left WVC − Right WVC)/(Left WVC + Right WVC)]. WVC have been shown to provide a better measure than voxel count alone, since binary voxel count alone ignores the value associated with a given voxel and therefore may not provide a representative measure of the actual contribution of each region (32, 33). Activation ARs closer to +1 indicate greater activity in the left MTL (separate analyses were conducted for hippocampus alone and hippocampus + parahippocampal gyrus) compared to right MTL, while activation ARs closer to −1 indicate greater activity in the right MTL compared to the left. If there was no activation above threshold in either the left or right ROI, then that data point was discarded for that patient. At the *t* > 1 threshold, there were five patient cases discarded (one RTLE, four LTLE) and four controls for the hippocampal analysis.

### Structural data analysis

Hippocampal volume was acquired using the multiple automatically generated templates (MAGeT) brain segmentation algorithm (34, 35)^2^. The MAGeT-Brain uses a set of atlas segmentations, which are propagated to a subset of a given sample to produce templates via non-linear registration. This produces a library of templates that are then propagated to each subject/target image and fused using a label fusion method. Using a template library generated from a subset of the sample, the segmentation takes advantage of the neuroanatomical variability in the subjects, thus providing the advantages of a multi-atlas segmentation approach, which has been shown to provide a closer estimate to manual tracing volumes compared to FSL FIRST and FreeSurfer (35). Hippocampal volumes acquired for patients were subsequently transformed into ARs according to the formula [(left volume − right volume)/(left volume + right volume)].

FreeSurfer (Martino Center for biomedical Imaging, Harvard-MIT, Boston, MA, USA)^3^ was used to find cortical thickness for the parahippocampal gyrus. Cortical thickness rather than cortical volume was used for the parahippocampal gyrus because the methods used by FreeSurfer tend to underestimate cortical volume due to its surface based measurement procedures. FreeSurfer has been previously described in detail (36) and has been assessed in terms of validity and accuracy (37–41). Preprocessing included intensity normalization, removal of non-brain tissue, Talaraich transformation, and segmentation of tissue into gray matter, white matter, and cerebral spinal fluid. Normalization and segmentation results were visually inspected to ensure accuracy and segmentation errors were amended through use of control points as recommended by the developers (36). To measure cortical thickness, the white and gray matter boundaries are identified and the distance between the two surfaces is calculated. The cortex is then automatically parcellated. Thickness is calculated for a structure based on this cortical parcelation. Structural ARs for the parahippocampal gyrus used the formula [(left thickness − right thickness)/(left thickness + right thickness)].

### Group comparison

One-way analysis of variance (ANOVA) was used to identify group differences for activation and structural ARs. Subsequent *t*-tests were used to look at pairwise group differences on neuropsychological factor scores, memory asymmetry scores, activation ARs, and structural ARs using SYSTAT 13 (Systat Software, Inc., Chicago, IL, USA). We also examined the relationship between our ARs and age, education, and disease duration to ensure that our subsequent analysis was not confounded by the influence of these factors using Pearson correlations.

### Neuroimaging measures and behavior

Mediation analysis was performed using linear regression in SYSTAT 13 (Systat Software, Inc., Chicago, IL, USA) and was conducted across patients with LTLE and RTLE to capture a greater variability of memory performance. The hippocampal activation ARs (acting as the predicted mediator) were regressed onto the hippocampal volume ARs (acting as the independent variable). The memory asymmetry scores (the dependent variable) were then regressed onto the hippocampal volume ARs. Finally, the memory asymmetry scores were regressed onto both the hippocampal activation ARs and the hippocampal volume ARs. We applied a Goodman test of significance as recommended by MacKinnon et al. (42). This test produces a *z*-score, which is used to test for significance. We also ran this analysis excluding those patients having abnormalities outside of the MTL.

## Results

### Demographics

There was no difference in age between the RTLE, the LTLE group, and the control group, *F*(35,2) = 2.56, *p* = 0.09. There was a significant group level difference in years of education, *F*(35,2) = 4.05, *p* < 0.05. Healthy controls had significantly more years of education than patients with RTLE, *t*(25) = 2.52, *p* < 0.05 and patients with LTLE, *t*(23) = 2.09, *p* < 0.05, but there was no difference between the two patient groups, *t*(25) = 0.68, *p* = 0.51. There was no difference in seizure duration between patients with RTLE and those with LTLE, *t*(25) = 0.37, *p* = 0.72. These demographic variables are summarized in Table 1.

### Neuropsychological performance

There was no significant difference between patients with LTLE and those with RTLE for the verbal memory factor, *t*(25) = 0.69, *p* = 0.5, or for the IQ factor, *t*(25) = 0.65, *p* = 0.52. Patients with RTLE had significantly lower visual memory factor scores than patients with LTLE, *t*(25) = 2.09, *p* < 0.05. The patient groups also differed significantly in memory asymmetry scores, *t*(25) = 2.62, *p* < 0.05, such that patients with RTLE showed greater verbal to visual memory asymmetry while patients with LTLE showed stronger visual than verbal memory.

### fMRI activation asymmetry

**Figure 2:**
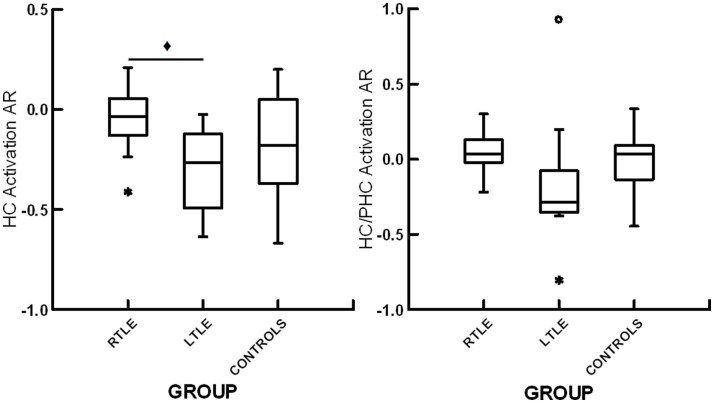
**Box plots displaying hippocampal activation asymmetry ratios (HC activation AR) and the joint hippocampal parahippocampal activation asymmetry ratios (HC/PHC activation AR) for patients with right temporal lobe epilepsy (RTLE), patients with left temporal lobe epilepsy (LTLE) and healthy controls (CONTROLS)**. *denotes outliers of >1.5 interquartile range from the median. ♦ denotes significant differences at *p* < 0.05.

All groups showed bilateral activation in occipital cortex, fusiform gyrus, parahippocampal gyrus, and hippocampus (*p* < 0.05, FDR corrected). A one-way ANOVA showed a trend toward significant differences among the RTLE, LTLE, and healthy control groups for hippocampal activation ARs, *F*(2,26) = 3.37, *p* = 0.05. Subsequent analysis revealed a significant difference between patients with RTLE and LTLE, *t*(20) = 3.01, *p* < 0.05, with the LTLE group showing greater right than left hippocampal activation whereas the RTLE group showed virtually symmetric activation. There were no significant differences between healthy controls and patients with RTLE, *t*(20) = 1.36, *p* = 0.19, or patients with LTLE, *t*(15) = 0.92, *p* = 0.37, for hippocampal asymmetry. For the combined hippocampal/parahippocampal asymmetry, there was no significant difference among the three groups, *F*(35,2) = 2.195, *p* = 0.13. A summary of these results can be seen in Figure 2. To allow for comparison to previous studies examining functional ARs with scene encoding, we have included a summary of effect sizes in Table 2. There was no relationship between functional ARs with age, sex, education, or disease duration (all *r* < 0.18, *p* > 0.05).

**Table 2:** **Summary of effect sizes (Cohen’s *d*) for hippocampal structural and functional asymmetry differences between patients with mTLE and healthy controls**.

		Barnett et al. (current)	Mechanic-Hamilton et al. (16)	Bigras et al. (20)
Functional AR	LTLE vs. controls	−0.5	−1.3	0.0
	RTLE vs. controls	0.6	0.2	0.8
Structural AR	LTLE vs. controls	−1.4	−1.2	–
	RTLE vs. controls	1.3	1.2	–

*AR, asymmetry ratio; LTLE, left medial temporal lobe epilepsy; RTLE, right medial temporal lobe epilepsy*.

### Structural asymmetry

**Figure 3:**
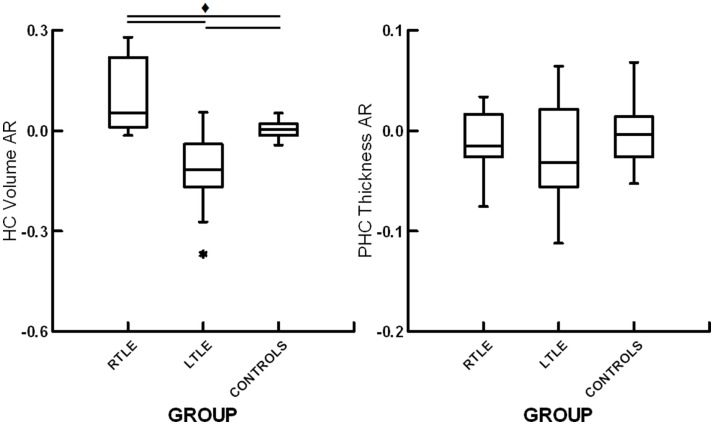
**Box plots displaying hippocampal volume asymmetry ratios (HC volume AR) and parahippocampal cortical thickness asymmetry ratios (PHC thickness AR) for patients with right temporal lobe epilepsy (RTLE), patients with left temporal lobe epilepsy (LTLE) and healthy controls (CONTROLS)**. * denotes outliers of >1.5 interquartile range from the median. ♦ denotes significant differences at *p* < 0.05.

A one-way ANOVA revealed a significant effect of group for hippocampal volume AR, *F*(2,35) = 17.9, *p* < 0.01. Subsequent contrasts revealed significant differences between healthy controls and patients with RTLE, *t*(25) = 3.19, *p* < 0.01, and between healthy controls and patients with LTLE, *t*(23) = 3.42, *p* < 0.01. There was also a significant difference between patients with RTLE and those with LTLE, *t*(25) = 5.03, *p* < 0.01. Both patient groups showed asymmetry skewed toward their unaffected hemisphere, with RTLE patients having relatively larger left hippocampi and LTLE patients having relatively larger right hippocampi. These results can be seen in Figure 3. To allow for comparison to a prior study examining hippocampal structural asymmetry by Mechanic-Hamilton et al. (16), we have included a summary of effect sizes in Table 2. There was no significant group difference for parahippocampal structural ARs, *F*(2,35) = 0.61, *p* = 0.55. There was also no relationship between structural ARs with age, education, sex, or disease duration (all *r* < 0.17, *p* > 0.05).

### Mediation analysis

**Figure 4:**
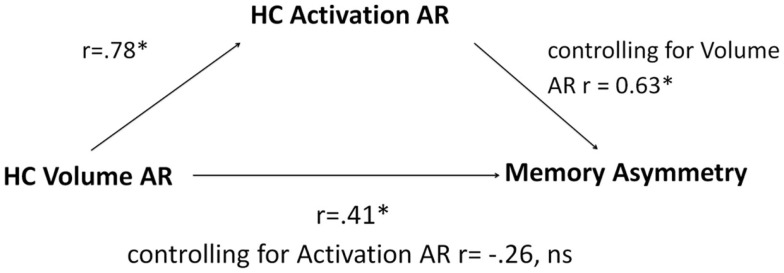
**A schematic depiction of the directional flow among the elements of the mediation model with accompanying correlation and partial correlation coefficients of regression equations**. HC, hippocampus; AR, asymmetry; ns, not significant, * denotes significant at *p* < 0.05.

Since only hippocampal ARs were able to segregate patients with LTLE from those with RTLE, we limited the mediation analysis to this measure. Linear regression revealed that hippocampal volume ARs predicted hippocampal activation ARs, *r* = 0.78, *p* < 0.001, establishing a link between the independent variable and the mediator. When used in isolation, hippocampal volume ARs also predicted memory asymmetry, *r* = 0.41, *p* < 0.05, establishing a link between the independent variable and the dependent variable. When both structural and functional ARs were included as predictors, hippocampal volume ARs were no longer significantly predictive (partial correlation of *r* = −0.26, *p* = 0.14), while hippocampal activation ARs were strongly predictive of memory asymmetry (partial correlation of *r* = 0.63, *p* < 0.01). Mediation analysis using the Goodman test revealed that the indirect path of the mediating relationship was significant, *z* = 3.01, *p* < 0.01, indicating that hippocampal activation AR mediates the effect of volume AR on memory asymmetry. The model is summarized in Figure 4.

Although all cases were classified as mTLE, five patients had abnormalities other than or in addition to MTS. Thus, we undertook the analysis again, excluding them, and found that our predictions remained significant. As in the original analysis, structural asymmetry predicted activation asymmetry (*r* = 0.62, *p* < 0.01) and memory asymmetry (*r* = 0.487, *p* < 0.05). When both functional and structural asymmetries were included in a regression model for memory asymmetry, however, hippocampal structural ARs no longer predicted memory (partial *r* = −0.23, *p* = 0.417), while hippocampal activation ARs remained predictive of memory asymmetry (partial *r* = 0.61, *p* < 0.05). The Goodman test revealed that the indirect path was significant, *z* = 2.27, *p* < 0.05.

## Discussion

A crucial question in translational neuroimaging research is how structural integrity may constrain functional activation and how they jointly may be used to predict cognitive capacity. Here, we address this by examining the relationship between MTL volume, functional activation during encoding, and behavioral measures of memory in patients with unilateral TLE. As expected from previous work, patients with LTLE had better visual than verbal memory, while patients with RTLE had better verbal than visual memory (5, 43). During the novel scene encoding task, as expected, most patients and controls activated bilateral MTL regions including the hippocampus. We identified significant differences in memory, activation, and volume asymmetry between patients with LTLE and those with RTLE, in agreement with past research (6, 16, 20, 43). While hippocampal volume asymmetry predicted pre-operative memory asymmetry in patients, consistent with past research (16), its ability to capture variance in memory was severely diminished when activation asymmetry was included in the model, suggesting the latter provides a more accurate estimate of functional capacity.

We found that patients with LTLE showed greater recruitment of their right hippocampus compared to their affected left MTL structures, which is consistent with several previous studies using this paradigm (16, 44). Patients with RTLE showed symmetrical activation in their MTL, consistent with Mechanic-Hamilton et al. (16) but different than the findings of Bigras et al. (20) where patients with RTLE showed greater left than right MTL. Our healthy controls showed a slight asymmetry toward right hippocampal activation. While neither patient group showed a significant difference from the healthy control group, it is important to note that both patient groups showed a bias toward their unaffected hippocampus in comparison to controls, an effect, which is consistent across the aforementioned studies (16, 20, 44, 45). Our results when using a larger region of interest encompassing the hippocampus and parahippocampus show more variability. Our three groups do not differ using this larger ROI to calculate asymmetry. Previous studies, however, found that when using a more encompassing region of interest, their results were largely unaffected (16, 20, 45). The differences between the current study and previous studies could potentially be due to the choice of fMRI task baseline, asymmetry calculation methods, or other factors. Where we contrasted novel and repeated scenes, Bigras et al. (20) and Mechanic-Hamilton et al. (16) used scrambled visual scenes against intact novel scenes. Our asymmetry calculations used a threshold of *t* > 1 whereas Mechanic-Hamilton et al. (16) used *t* > 0 and Bigras et al. (20) used a median split threshold. As the field moves toward adoption of fMRI in clinical contexts, identification of the paradigm and processing choices that are most sensitive to memory performance and memory change is becoming increasingly important.

Our structural results, demonstrating smaller hippocampal volume in the affected hemisphere compared to the unaffected hemisphere, are also consistent with previous research (16, 20, 46–48). Reduced volumes in the affected hemisphere are thought to be due to hippocampal pathology particularly hippocampal sclerosis that is often seen in mTLE (49). We did not see asymmetric reductions in parahippocampal cortices of patients. FreeSurfer parcelation provides an average cortical thickness value for the parahippocampal gyrus encompassing both anterior and posterior regions. Previous findings of reduced MTL cortex in TLE have been limited to entorhinal cortices (47, 50, 51) and the coarse parcelation provided by FreeSurfer may not be sensitive to this effect.

Our principal aim was to characterize the relationship between structure, functional activation, and memory. Our finding, that activation asymmetry mediates the relationship between structural asymmetry and memory performance, suggests that structural integrity may identify the necessary substrate for behavior but that its influence on performance is more adequately expressed in terms of the capacity to engage that substrate appropriately. Previous research in TLE has shown that reduced volume in the ipsilateral hippocampus is associated with lower peak activation there (20), suggesting that damage to this structure constrains its ability to functionally activate. We found similar results in terms of asymmetry, in that patients with larger left than right hippocampal volumes also demonstrated greater left than right activation. While these two indices of hippocampal integrity were correlated with one another and with memory performance, these relationships were asymmetric in that activation captured more of the variance across patients in terms of memory when both were considered in a mediation model. These results indicate an indirect role for structural integrity in explaining memory performance, a relationship that was also demonstrated using functional connectivity of the default mode network by McCormick et al. (52). This suggests that in cases of minimal volume asymmetry, those patients who are unable to activate their epileptogenic hippocampus will experience material-specific memory impairments.

In the context of material-specific memory impairments, asymmetrical functional activation away from the affected hemisphere has been interpreted as demonstrating an inability of the contralateral hippocampus to support residual memory function (20, 45, 53). Indeed, where activation in the “healthy” MTL has been examined discretely as a predictor of memory for the “non-specialized” type of memory (e.g., right MTL activation for verbal memory), there has generally not been a significant relationship demonstrated. Of interest, our previous study of resting-state connectivity between the posterior cingulate cortex and hippocampus (54) did show some evidence compatible with functional reserve or compensation in that stronger contralateral connectivity was associated with less post-operative decline. This may indicate that connectivity provides a more sensitive marker of the potential contribution of both MTL regions to memory.

The other studies that have examined activation asymmetry have used individual test scores (or change scores on specific tests) as the outcome variable. While several of these have shown adequate correlations between these measures (15, 16, 20, 45), we elected to use composite factor scores to evaluate verbal and visual memory rather than individual memory tests. These composite scores have been shown to predict post-operative change (22) and are related to network level functional connectivity (52). Using composite scores, we also reduced the need to perform large numbers of statistical tests, which would inflate the likelihood of committing a type I error.

This study has several limitations. First, we did not record any behavioral measure during scanning to ensure that subjects were attending appropriately to stimuli. A subset of our sample had to be dropped due to insufficient hippocampal activation, which may have been due to disengagement. Among the participants that we retained for the analysis, we are confident in task engagement based upon the reliable MTL activation observed that was consistent with previous literature. Second, all patients were taking anticonvulsant medication during the scanning and neuropsychological procedures. This is an unavoidable circumstance in research with TLE, but it does comport with the clinical context in which decisions that fMRI may support are made. Our sample included five patients with lesions other than or in addition to MTS. However, the disorder of mTLE is considered as heterogeneous (55) with MRI evidence of focal cortical dysplasia and low-grade tumors being not uncommon in the MTL (56). With respect to remote lesions, data suggest it can be difficult to distinguish individuals with exclusive MTS from those with developmental lesions in terms of surgical outcome (56). All five of these patients were considered to be surgical candidates for standard anterior temporal lobe resections based on consensus conference, and four of the five patients underwent this surgery at the time of this report. Excluding them did not change any of the major findings. New advances in fMRI acquisition protocols such as multiband EPI sequences (57) are allowing for better signal to noise ratio through increased temporal and spatial resolution. Our current study did not apply these new techniques and future studies employing these techniques may be able to better characterize regions within the hippocampus specific to these memory effects. Finally, we acknowledge that our sample size was relatively small. Because of this limitation, we also examined effect sizes for variables of interest and ours are moderate to high (see Table 2). Nonetheless, the limited sample may have diminished our ability to identify relationships with clinical variables such as age of onset and to detect a significant difference between left and right TLE patients on the verbal memory composite. Thus, while we feel this cohort was representative of the mTLE population, based on neuropsychological performance and MRI asymmetry measures, and have confidence in the positive results reported, we acknowledge that future research with a larger sample is needed to derive strong conclusions about relationships amongst these variables.

In conclusion, functional integrity measured by hippocampal activation asymmetry mediates the relationship between structural measure of integrity and memory performance. These findings support the call for the continued investigation of fMRI memory lateralization in characterizing functional adequacy of the hippocampus in mTLE, particularly as they seem to be key to explaining the link between structural degradation and memory capacity in this population.

## Conflict of Interest Statement

The authors declare that the research was conducted in the absence of any commercial or financial relationships that could be construed as a potential conflict of interest.

## Footnotes

^1^http://www.bion.de/eng/MARINA.php

^2^https://github.com/pipitone/MAGeTbrain

^3^http://surfer.nmr.mgh.harvard.edu
